# Comparison of the Health Status of Vegetarians and Omnivores Based on Biochemical Blood Tests, Body Composition Analysis and Quality of Nutrition

**DOI:** 10.3390/nu15133038

**Published:** 2023-07-05

**Authors:** Paulina Jedut, Paweł Glibowski, Michał Skrzypek

**Affiliations:** 1 Department of Biotechnology, Microbiology and Human Nutrition, University of Life Sciences in Lublin, 8 Skromna St., 20-704 Lublin, Poland; paulinajedut1@wp.pl; 2 Faculty of Health Sciences, Vincent Pol University in Lublin, 20-816 Lublin, Poland; mskr@poczta.onet.pl

**Keywords:** vegetarian diet, biochemical analysis of blood, BIA

## Abstract

Many vegetarians are motivated by the health aspect of starting a plant-based diet. This diet can offer many health benefits. The study aimed to check whether people on a vegetarian diet are in good health, have a good nutritional intake, and follow the principles of healthy eating compared with omnivores. Twenty-two vegetarians on a vegetarian diet for more than five years and 22 omnivores aged 18–45 were interviewed. Each of them was given a food questionnaire, body composition analysis (BIA), and biochemical blood analysis, and their 7-day diet was analyzed. Polish vegetarians exhibit similar health statuses and tend towards better health than omnivores. They have an adequate body composition. Biochemical blood analysis showed no significant differences in blood parameters between vegetarians and omnivores, despite specific deficiencies such as vitamin B_12_, vitamin D and elevated homocysteine levels in vegetarians. They have a better nutritional status and follow good dietary principles. However, they were more likely to consume alcohol, add salt to their meals and sweeten hot drinks. In addition, the lifestyle of vegetarians can be described as healthier, as they are more likely to engage in leisure-time physical activity and get enough sleep.

## 1. Introduction

Vegetarian diets, based on the complete exclusion of meat and fish from the daily menu and, depending on the level of restrictions, diets limiting the consumption of animal products, are gaining more and more popularity. According to a report on the nutrition of Poles, already 10% of Poles between the ages of 18 and 65 are vegetarians, and 6% are vegans [1]. As the reason for starting a plant-based diet, vegetarians indicate ethics and health aspects [2,3]—for a good cause, because choosing a restrictive vegetarian diet can offer many health benefits. The American Dietetic Association (ADA) 2009 stated that properly balanced vegetarian diets, including vegan, are healthy, meet nutritional needs, and can offer health benefits in preventing and treating certain diseases [4]. A meta-analysis, which included 13 cohort studies with 844,157 participants, concluded that vegetarian diets are associated with a 15% reduced risk of cardiovascular disease and a 21% reduced risk of ischemic heart disease [5]. American researchers came to similar conclusions. Their meta-analysis of 8 studies confirmed that a vegetarian diet compared with a non-vegetarian diet was associated with a 30% reduced mortality risk due to ischemic disease [6]. Also, diabetes was 46% less common among people on plant-based diets, particularly among vegans (49%) than omnivores [7]. The above condition and the improvement of glycemic control are most likely due to the greater consumption of wholegrain products rich in fiber, legumes, vegetables, and fruits and reduced consumption of meat products and products of animal origin, a source of saturated fatty acids [8]. It is generally known that eating fewer meat products and more plant-based foods may be associated with improved lipid profile scores, lower triglycerides (TG), low-density lipoproteins (LDL), reduced glucose levels as well as lower Body Mass Index (BMI), and lower Waist Circumference (WC) [9,10]. Another meta-analysis of cohort studies conducted among adults showed that using a plant-based diet was associated with lower mortality in people with chronic kidney disease. Based on an estimated 5-year mortality of 17% in people with chronic kidney disease, the risk difference with vegetarian diet patterns compared with other dietary patterns was 46 fewer deaths per 1000 people [11]. A study was also conducted with vegetarians to assess their overall health. The study included 1209 adult vegetarians from Poland and the USA. The survey results showed that most respondents felt an improvement in their health after giving up meat consumption. The progress was mainly due to the reduction of gastroenterological symptoms such as flatulence, heartburn, nausea, and diarrhea [12].

It should be noted that despite a plant-based diet’s broad health benefits, a lack of appropriate balance may be associated with adverse health outcomes. The sudden transition from a non-vegetarian diet to a vegetarian diet can be related to, among others, noticeable skin changes [12]. In addition, a vegetarian diet can lead to nutritional deficiencies, including vitamins and minerals. The most common deficiencies are protein, omega-3 fatty acids, vitamin D, B12, iron, calcium, and zinc [13].

As mentioned above, studies confirm the health impact of a vegetarian diet on humans, checking the effect of a vegetarian diet on blood biochemical parameters or anthropometry. To our knowledge, no studies have compared the general health of vegetarians to omnivores, combining the analysis of blood biochemistry results, bioelectrical impedance analysis results, and dietary assessment. Moreover, none have been conducted on Polish subjects.

This study aimed to check whether people following a vegetarian diet for a long time are healthier than omnivores.

The research hypothesis assumed that biochemical blood tests and body composition analysis results would be more favorable in people who follow a vegetarian diet. In addition, we wanted to show that vegetarians eat more following the principles of healthy eating than omnivores. This also means that vegetarians consume more vegetables, fruits, and other plant-based products and fewer animal and highly processed products than omnivores.

## 2. Materials and Methods

The study was conducted according to the guidelines of the Declaration of Helsinki and approved by the Ethics Committee of the Medical University of Lublin (decision number: KE-0254/216/2020).

### 2.1. The Test Group

To select the study group, which consisted of Poles following a vegetarian diet, an advertisement was posted on social networking sites. The volunteers were asked to complete a questionnaire, which helped qualify them for participation in the study. The criteria for joining the study were as follows: on a vegetarian diet for more than five years, aged 18–45. In addition, the study protocol assumed that people diagnosed with type I diabetes, epilepsy, pregnant women, people with an implanted pacemaker or cardioverter, defibrillator, and metal implants, excluding dental implants, were excluded from participation in the study. The control group consisted of omnivores selected based on the “pair matching” principle—i.e., according to individual compatibility in setting the control group to the study group regarding matching variables: gender, age, place of residence, and education. Individual matching was intended to indicate differences more accurately in health and diet. Ultimately, 22 vegetarians and 22 omnivores, both women and men, participated in the study.

### 2.2. Outcome Measurements

An individual meeting was held with each participant of the study, during which anthropometric measurements were made, a nutritional interview was conducted, blood analysis was ordered in an external laboratory, and a nutritional diary was ordered. Body height was measured in a standing position using the SECA 216 (seca GmbH & Co. KG., Hamburg, Germany) wall-mounted stadiometer with an accuracy of 0.1 cm. We measured the waist circumference with SECA 201 metric tape. The SECA mBCA515 analyzer measured body weight with an accuracy of 0.1 kg. BMI was used to assess relative body weight—a popular indicator for measuring, for example, obesity—and nutritional and health status, applicable to both women and men [14]. Body composition was measured using the SECA mBCA515 analyzer (Seca GmbH & Co. KG., Hamburg, Germany) using the bioelectrical impedance analysis (BIA) method (eight-point). This method is often used to assess body composition in dietary practice [15].

To analyze the study participants’ diet, a nutritional interview was conducted with questions from the KOMPAN questionnaire [16]. In addition, they were asked to keep food diaries for seven days, where the amount of food consumed had to be recorded. The diaries were analyzed using Aliant 2.0 software (Anmarsoft, Gdańsk, Poland). The results of the analysis were compared with the Dietary Reference Value of EFSA (European Food Safety Authority, Parma, Italy) [17], which helped identify nutritional deficiencies of macronutrients as well as minerals and vitamins.

Blood samples were collected (after fasting) and tested by an external diagnostic laboratory for glucose, calcium, total cholesterol (Total-chol), low-density lipoprotein (LDL), high-density lipoprotein (HDL), triglycerides (TG), total protein, albumin, iron, ferritin, vitamin B_12_, vitamin D_3_, homocysteine, complete blood count.

### 2.3. Statistical Analysis

Statistical analysis was performed using Statistica software (v13.3, StatSoft, Kraków, Poland). Data expressed on a qualitative scale were presented as the number and percentage of the sample. The Chi-squared test (χ^2^) was used to compare the relationships between variables expressed in the qualitative scale. Data expressed on a quantitative scale were presented as a mean with standard deviation (SD). Depending on the result of the Shapiro-Wilk test (assessment of compliance with the normal distribution), the student’s *t* test or Mann-Whitney test was used. Results were considered statistically significant when *p* < 0.05.

## 3. Results

The study involved 22 subjects following one type of vegetarian diet (lacto-ovo-vegetarian, lactovegetarian, ovovegetarian, and vegan) and 22 omnivores. In both groups, the vast majority were women (82%). The average age of the subjects was 30 ± 7.56 years. Most respondents lived in cities with over 100,000 inhabitants (64%) and had higher education (77%).

### 3.1. Survey Results

Individual meetings were held with all the vegetarians and omnivores, during which they were questioned about their lifestyles and eating habits. The results of the surveys are presented in Table 1, Table 2, Table 3, Table 4, Table 5, Table 6, Table 7, Table 8 and Table 9. Table 1 compares the eating habits of both groups, looking at the number of meals consumed, the regularity of eating meals, types of spreadable fat used, frying fat, and the habit of sweetening hot beverages and adding salt to food. The data shows that both vegetarians and omnivores consume a similar number of meals. Most subjects from both groups ate four meals, and only one person from the omnivores group ate only two meals daily. There was no statistically significant difference between the groups (*p* > 0.05). Most vegetarians and omnivores eat only certain meals regularly, and vegetarians are much less likely to eat all their meals at regular times. There was no statistically significant difference between the groups (*p* > 0.05). Regarding snacking between meals, vegetarians were less likely to snack than omnivores. This difference is statistically significant (*p* < 0.05). Among the respondents, only three vegetarians never snacked during the day.

Both vegetarians and omnivores use butter and margarine as spreads, and other types of fat are less common in both groups. The exact number of subjects in both groups indicated they did not use any spreadable fat, and the results were not statistically significant (*p* > 0.05). Vegetable oil, including olive oil, was the most frequently chosen fat for frying by both groups. At the same time, the group of vegetarians selecting this type of fat was more numerous compared to omnivores. Also, in the case of butter, which ranked second in preference for frying food, vegetarians were likelier to choose this type of fat than omnivores. A small percentage of vegetarians fry in coconut oil, while one omnivore chose lard. These differences were not statistically significant (*p* > 0.05).

In both groups, most respondents do not sweeten hot drinks. This also shows that vegetarians sweeten hot drinks more often than omnivores. These differences were statistically significant (*p* < 0.05).

Vegetarians have a habit of adding extra salt to their dishes. More than 22% of subjects following a plant-based diet declared that they add salt to most dishes, while none of the omnivores presented this habit. Both vegetarians and omnivores often use salt only in some cases. The analysis showed a statistically significant difference in salt addition between these groups (*p* < 0.05). The vegetarian group was characterized by more subjects who did not give up adding salt to their diet than omnivores.

Table 2 presents the number and percentage of the surveyed subject in particular groups who consume various food products with a specific frequency. White bread was the most popular among vegetarians, eaten several times daily. Omnivores more often opted for wholemeal bread. Vegetarians consumed less white rice, plain pasta, and barley groats than omnivores. The consumption of oatmeal, whole grain pasta, and buckwheat groats was comparable in both groups. The results of the above analyzes were not statistically significant (*p* > 0.05). The consumption of legumes differed, with vegetarians much more likely to eat them (*p* < 0.05). A similar situation occurred with the consumption of canned vegetables and pickles. Vegetarians were more likely to opt for them compared to omnivores. These results were statistically significant (*p* < 0.05). Differences in the consumption of fruit, vegetables, and potatoes by both study groups were not statistically significant (*p* > 0.05), although these products were consumed by vegetarians slightly more often. More powered and canned soups were consumed by vegetarians (*p* > 0.05); however, this was statistically insignificant. Sweets, fast food, and fried meals were more likely to be eaten by omnivores compared to vegetarians. Despite the difference, the results were not statistically significant (*p* > 0.05).

During the survey, participants were also asked about the frequency of fluid intake. The results are presented in Table 3. Consumption of water and fruit juices was comparable in both groups (*p* > 0.05). Vegetable or vegetable and fruit juices were more popular among vegetarians. As many as 70% of omnivores do not drink this type of juice. Differences in frequency of consumption were statistically significant (*p* < 0.05). Differences in the consumption of sweet carbonated and non-carbonated drinks and energy drinks by vegetarians and omnivores were statistically insignificant (*p* > 0.05). Despite this, omnivores tend to consume these drinks more frequently. Statistical analysis showed a significant difference in alcohol consumption (*p* < 0.05). Alcohol consumption was reported to be more frequent among vegetarians. One vegetarian drank alcohol daily.

**Table 2:** Comparison of the frequency of consumption of food products by vegetarians and omnivores.

Products [*n* (%)]	Vegetarians (*n* = 22)						Omnivores (*n* = 22)						*p* Value
	Women (*n* = 18), Men (*n* = 4)						Women (*n* = 18), Men (*n* = 4)						
	Several Times a Day	Once a Day	Several Times a Week	Once a Week	1–3 Times a Month	Never	Several Times a Day	Once a Day	Several Times a Week	Once a Week	1–3 Times a Month	Never	
White bread	4 (18.2)	2 (9.1)	9 (40.9)	2 (9.1)	4 (18.2)	1 (4.6)	4 (18.2)	7 (31.8)	7 (31.8)	2 (9.1)	2 (9.1)	0 (0)	0.3851
Whole grain bread	3 (13.6)	4 (18.2)	6 (27.3)	2 (9.1)	4 (18.2)	3 (13.6)	1 (4.6)	1 (4.6)	8 (36.4)	2 (9.1)	8 (36.4)	2 (9.1)	0.4380
White rice, plain pasta, small groats	0 (0)	0 (0)	2 (9.1)	3 (13.6)	3 (13.6)	1 (4.6)	0 (0)	0 (0)	16 (72.7)	1 (4.6)	4 (18.2)	1 (4.6)	0.3702
Oatmeal, whole grain pasta, coarse grain groats	0 (0)	2 (9.1)	9 (40.9)	5 (22.7)	4 (18.2)	2 (9.1)	0 (0)	3 (13.6)	10 (45.5)	5 (22.7)	3 (13.6)	1 (4.6)	0.9466
Legumes	2 (9.1)	2 (9.1)	10 (45.5)	2 (9.1)	4 (18.2)	0 (0)	0 (0)	1 (4.6)	4 (18.2)	0 (0)	13 (59.1)	4 (18.2)	0.0009 ^$^
Potatoes	0 (0)	4 (18.2)	9 (40.9)	5 (22.7)	6 (27.3)	0 (0)	0 (0)	0 (0)	7 (31.8)	6 (27.3)	9 (40.9)	0 (0)	0.2935
Fruit	6 (27.3)	2 (9.1)	10 (45.5)	2 (9.1)	2 (9.1)	0 (0)	4 (18.2)	5 (22.7)	9 (40.9)	3 (13.6)	1 (4.6)	0 (0)	0.6762
Vegetables	14 (63.6)	2 (9.1)	3 (13.6)	2 (9.1)	1 (4.6)	0 (0)	11 (50)	4 (18.2)	5 (22.7)	2 (9.1)	0 (0)	0 (0)	0.5692
Canned vegetables, pickles	2 (9.1)	2 (9.1)	9 (40.9)	6 (27.3)	5 (22.7)	0 (0)	0 (0)	0 (0)	5 (22.7)	3 (13.6)	10 (45.5)	4 (18.2)	0.0159 ^$^
Ready powdered soups, soups in cans	0 (0)	0 (0)	2 (9.1)	2 (9.1)	5 (22.7)	13 (59.1)	0 (0)	0 (0)	1 (4.6)	0 (0)	5 (22.7)	16 (72.7)	0.3308
Fast foods	0 (0)	0 (0)	4 (18.2)	1 (4.6)	11 (50)	5 (22.7)	0 (0)	0 (0)	2 (9.1)	7 (31.8)	11 (50)	2 (9.1)	0.0762
Sweets	1 (4.6)	1 (4.6)	8 (36.4)	5 (22.7)	4 (18.2)	3 (13.6)	0 (0)	5 (22.7)	10 (45.5)	2 (9.1)	4 (18.2)	1 (4.6)	0.2285
Fried meals	0 (0)	1 (4.6)	6 (27.3)	6 (27.3)	8 (36.4)	1 (4.6)	0 (0)	2 (9.1)	8 (36.4)	5 (22.7)	4 (18.2)	3 (13.6)	0.5374

Note: Data are presented as number (percentage) of participants, ^$^ *p*-value—statistically significant difference between vegetarians and omnivores, *p* < 0.05.

**Table 3:** Comparison of the frequency of consumption of drinks by vegetarians and omnivores.

Drinks [*n* (%)]	Vegetarians (*n* = 22)						Omnivores (*n* = 22)						*p* Value
	Women (*n* = 18), Men (*n* = 4)						Women (*n* = 18), Men (*n* = 4)						
	Several Times a Day	Once a Day	Several Times a Week	Once a Week	1–3 Times a Month	Never	Several Times a Day	Once a Day	Several Times a Week	Once a Week	1–3 Times a Month	Never	
Water	17 (77.3)	2 (9.1)	1 (4.6)	1 (4.6)	1 (4.6)	0 (0)	16 (72.7)	1 (4.6)	4 (18.2)	0 (0)	1 (4.6)	0 (0)	0.4504
Fruit juices	1 (4.6)	1 (4.6)	1 (4.6)	8 (36.4)	4 (18.2)	7 (31.8)	0 (0)	2 (9.1)	2 (9.1)	3 (13.6)	9 (40.9)	6 (27.3)	0.2627
Vegetable or vegetable and fruit juices	0 (0)	0 (0)	4 (18.2)	6 (27.3)	4 (18.2)	8 (36.4)	0 (0)	0 (0)	2 (9.1)	0 (0)	5 (22.7)	15 (68.2)	0.0103 ^$^
Sweetened carbonated and still drinks	0 (0)	1 (4.6)	1 (4.6)	3 (13.6)	8 (36.4)	9 (40.9)	0 (0)	0 (0)	2 (9.1)	1 (4.6)	10 (45.5)	9 (40.9)	0.5586
Energy drinks	0 (0)	0 (0)	1 (4.6)	0 (0)	3 (13.6)	18 (81.8)	0 (0)	0 (0)	0 (0)	2 (9.1)	4 (18.2)	16 (72.7)	0.2195
Alcohol	0 (0)	1 (4.6)	4 (18.2)	3 (13.6)	9 (40.9)	5 (22.7)	0 (0)	0 (0)	0 (0)	1 (4.6)	18 (81.8)	3 (13.6)	0.0211 ^$^

Note: Data are presented as number (percentage) of participants, ^$^ *p*-value—statistically significant difference between vegetarians and omnivores, *p* < 0.05.

The lifestyle habits of both groups are presented in Table 4. Four factors were analyzed: cigarette smoking, hours of sleep on weekdays and weekends, and time spent watching TV and working in front of the computer. There were no significant differences between the groups in terms of these habits.

**Table 4:** Lifestyle habits comparison of vegetarians and omnivores.

Lifestyle Habits [*n* (%)]	Vegetarians (*n* = 22)	Omnivores (*n* = 22)	*p* Value
	Women (*n* = 18), Men (*n* = 4)	Women (*n* = 18), Men (*n* = 4)	
***Smoking cigarettes***			
Yes	4 (18.2)	2 (9.1)	0.6604
No	18 (81.8)	20 (90.9)	
***Hours of sleep on weekdays***			
Nine and more hours/day	2 (9.1)	0 (0)	0.2065
7 or 8 h/day	14 (63.6)	17 (77.3)	
Six or less hours/day	6 (27.3)	5 (22.7)	
***Hours of sleep on the weekend***			
Nine and more hours/day	10 (45.5)	6 (27.3)	0.3154
7 or 8 h/day	9 (40.9)	14 (63.6)	
Six or less hours/day	3 (13.6)	2 (9.1)	
***Watching TV and working in front of the computer***			
>10 h	2 (9.1)	3 (13.6)	0.9869
8–10 h	7 (31.8)	6 (27.3)	
6–8 h	5 (22.7)	5 (22.7)	
4–6 h	2 (9.1)	3 (13.6)	
2–4 h	2 (9.1)	2 (9.1)	
<2 h	4 (18.2)	3 (13.6)	

Note: Data are presented as the number (percentage) of participants.

The survey questions also looked at physical activity. Thanks to this, information was obtained on two categories of physical activity: during work and leisure time. The results show no significant differences between the groups regarding physical activity at work (*p* > 0.05). Despite this, the table indicates that vegetarians were more active at work. In the case of differences between the groups regarding physical activity in leisure time, a statistically significant relationship was observed (*p* < 0.05). Vegetarians accounted for more subjects who practised physical activity in their spare time. In comparison, the omnivores group had a higher rate of subjects who practiced little physical activity in their spare time.

**Table 5:** Comparison of physical activity levels among vegetarians and omnivores throughout the day.

Physical Activity [*n* (%)]	Vegetarians (*n* = 22)	Omnivores (*n* = 22)	*p* Value
	Women (*n* = 18), Men (*n* = 4)	Women (*n* = 18), Men (*n* = 4)	
***During the day at work***			
Big *	3 (13.6)	2 (9.1)	0.3154
Moderate **	10 (45.5)	6 (27.3)	
Little ***	9 (40.9)	14 (63.6)	
***In spare time***			
Big ^#^	5 (22.7)	3 (13.6)	0.0007 ^$^
Moderate ^##^	9 (40.9)	8 (36.4)	
Little ^###^	8 (36.4)	11 (50)	

Note: Data are presented as number (percentage) of participants, ^$^ *p*-value—statistically significant difference between vegetarians and omnivores, *p* < 0.05, *—about 70% of the time in motion or strenuous physical work **—about 50% of the time sitting and about 50% of the time moving ***—more than 70% of the time sitting ^#^—cycling, running, gardening or gardening and other sporting, recreational activities requiring more than 3 h of physical effort per week, ^##^—walking, cycling, gymnastics, gardening or other light physical activity performed for 2–3 h a week, ^###^—mostly sitting, watching TV, reading the press, books, light housework, walking 1–2 h a week.

We also decided to find out how the surveyed subjects perceive their diet and how they assess their health in comparison to their peers. Table 6 presents the differences in the subjective health and nutrition assessment of vegetarians and omnivores. The results show significant differences (*p* < 0.05) between the groups in assessing health compared to peers. A higher percentage of vegetarians rated their health as better. On the other hand, the omnivores group had a higher percentage of subjects who assessed their health as the same as subjects of the same age. In terms of subjective assessment of nutrition, no significant differences were found between the groups (*p* > 0.05). Both in the group of vegetarians and in the group of omnivores, most subjects considered their diet good.

**Table 6:** Comparison of the subjective assessment of vegetarians and omnivores in terms of their health and diet.

Subjective Assessment [*n* (%)]	Vegetarians (*n* = 22)	Omnivores (*n* = 22)	*p* Value
	Women (*n* = 18), Men (*n* = 4)	Women (*n* = 18), Men (*n* = 4)	
***Health compared to peers***			
Better	8 (36.4)	2 (9.1)	0.0162 ^$^
Same	9 (40.9)	18 (81.8)	
Worse	5 (22.7)	2 (9.1)	
***Way of nutrition***			
Very good	2 (9.1)	3 (13.6)	0.3385
Good	13 (59.1)	10 (45.5)	
Bad	7 (31.8)	7 (31.8)	
Very bad	0 (0)	2 (9.1)	

Note: Data are presented as number (percentage) of participants, ^$^ *p*-value—statistically significant difference between vegetarians and omnivores, *p* < 0.05.

### 3.2. Analysis of Food Diaries

Table 7 presents the results obtained from the analysis of all subjects’ food diaries, divided by the type of diet used. The presented data are the mean values and standard deviations of a seven-day energy intake and various nutrients. The average energy intake by both groups was similar and showed no statistically significant difference (*p* > 0.05). Protein intake by vegetarians was lower compared to omnivores, but this difference was also not statistically significant (*p* > 0.05). Fat, carbohydrate, and fiber intake were higher among vegetarians than omnivores. In the case of fiber, the difference was statistically significant (*p* < 0.05), while in the case of fat and carbohydrates, the difference was insignificant (*p* > 0.05). There was also a statistically significant difference in the consumption of phosphorus and iodine, with omnivores consuming more than vegetarians (*p* < 0.05). In turn, copper intake was higher among vegetarians (*p* < 0.05). There were no statistically significant differences in the intake of other minerals (*p* > 0.05). Regarding vitamins, vegetarians had significantly more vitamin A in their diet than omnivores, which was statistically significant (*p* < 0.05). There was also a considerable difference in the intake of vitamins D, riboflavin, and B12. Omnivores consumed more of these compared to vegetarians (*p* < 0.05). In the case of other vitamins, there were no statistically significant differences between the two groups (*p* > 0.05). SFA and MUFA intake levels did not differ statistically significantly (*p* > 0.05) between vegetarians and omnivores, although it is worth noting that vegetarians presented less SFA and more MUFA. The difference in PUFA intake was statistically significant. Vegetarians consumed more fatty acids of this type compared to omnivores (*p* < 0.05). Omnivores drank more water and consumed sucrose and fructose, while vegetarians ate more glucose. However, these differences between the two groups were not statistically significant (*p* > 0.05). Omnivores absorbed much more cholesterol from food than vegetarians; this difference was statistically significant (*p* > 0.05).

**Table 7:** The seven-day average intake of energy and selected nutrients among vegetarians and omnivores.

Nutrients [$\overset{-}{x}$ ± SD]	Vegetarians (*n* = 22)	Omnivores (*n* = 22)	*p* Value
	Women (*n* = 18), Men (*n* = 4)	Women (*n* = 18), Men (*n* = 4)	
Energy (kcal)	1962.9 ± 480.3	1928.4 ± 389.7	0.7954
Total proteins (g)	75.4 ± 36.3	84.4 ± 26.9	0.2178
Fat (g)	76.4 ± 31.2	71.6 ± 13.2	0.5063
Total carbohydrates (g)	264.6 ± 66.5	253.7 ± 60.1	0.5698
Fiber (g)	37.9 ± 12.3	28.6 ± 8.5	0.0058 ^$^
Sodium (mg)	2711.7 ± 1007.2	2478.6 ± 626.6	0.3619
Potassium (mg)	3317.8 ± 984.1	3514 ± 1056.1	0.5273
Calcium (mg)	747.7 ± 425.4	776.6 ± 289.7	0.5652
Phosphorus (mg)	1168.9 ± 331.4	1426.5 ± 431.4	0.0318 ^$^
Magnesium (mg)	443.5 ± 140.6	384.5 ± 112.8	0.1326
Iron (mg)	15.5 ± 4	14.1 ± 3.9	0.2704
Zinc (mg)	10.3 ± 2.9	10.9 ± 2.9	0.4847
Copper (mg)	2.2 ± 0.7	1.5 ± 0.5	0.0008 ^$^
Iodine (µg)	46.3 ± 45.9	48.9 ± 15.4	0.0183 ^$^
Vitamin A (µg)	2017.1 ± 1203.4	1295.6 ± 486.7	0.0151 ^$^
Vitamin D (µg)	1 ± 0.6	5.8 ± 10.4	0.0000 ^$^
Vitamin E (mg)	15.2 ± 10	12.8 ± 3.5	0.8053
Thiamin (B_1_) (mg)	1.3 ± 0.3	1.5 ± 0.4	0.0703
Riboflavin (B_2_) (mg)	1.2 ± 0.4	1.9 ± 0.6	0.0002 ^$^
Vitamin B_6_ (mg)	1.9 ± 0.7	2.3 ± 0.7	0.0700
Folates (µg)	405.1 ± 129.1	415.7 ± 122.6	0.7830
Vitamin B_12_ (µg)	0.7 ± 0.7	3.6 ± 1.4	0.0000 ^$^
Vitamin C (mg)	207.3 ± 129.7	189.8 ± 88.8	0.6040
SFA (g)	19.3 ± 11.4	21.5 ± 5.9	0.1300
MUFA (g)	25.6 ± 9.8	28.7 ± 6.7	0.2188
PUFA (g)	18.9 ± 10	12.4 ± 3.2	0.0265 ^$^
Water (mL)	1456.4 ± 495.4	1596.2 ± 444.6	0.3303
Cholesterol (mg)	160.3 ± 143	362.1 ± 112.3	0.0000 ^$^
Sucrose (g)	28.5 ± 17.5	29.3 ± 12.2	0.7513
Glucose (g)	14.4 ± 7	12.2 ± 3.9	0.2007
Fructose	15.3 ± 7.7	16.1 ± 5.3	0.7029

Note: Each value is the mean ± standard deviation, ^$^ *p*-value—statistically significant difference between vegetarians and omnivores, *p* < 0.05, Abbreviations SFA—saturated fatty acids, MUFA—monounsaturated fatty acids, PUFA—polyunsaturated fatty acids.

Their seven-day average intake was compared to the EFSA nutritional standards for individual ingredients. Table 8 presents the results in the AR (according to references) and BR (below references) columns, divided by the type of diet used. Fiber intake was statistically different between the two groups. Most vegetarians consumed adequate amounts of fiber; in the case of omnivores, only half of the group (*p* < 0.05) did so. A similar percentage of vegetarians and omnivores consumed adequate amounts of potassium, so the difference was not statistically significant (*p* > 0.05). The case was identical with phosphorus, magnesium, zinc, vitamin A, folic acid, and vitamin C—most vegetarians and omnivores ate them in adequate amounts. Therefore, the differences between the groups were statistically insignificant (*p* > 0.05). Regarding calcium, iron, copper, iodine, and vitamin D, vegetarians and omnivores had trouble getting enough. Over half of vegetarians and omnivores consumed insufficient amounts (*p* > 0.05). Slightly more omnivores had adequate vitamin E than vegetarians (*p* > 0.05). The difference between the number of vegetarians consuming the recommended amount of riboflavin according to the nutritional standard and the number of omnivores was statistically significant. Most vegetarians did not get adequate amounts of vitamin B2 from food, while over 60% of omnivores had adequate amounts (*p* < 0.05). The intake of vitamin B6 looks similar, where fewer vegetarians consume it in sufficient quantities compared to omnivores. However, this difference was not statistically significant (*p* > 0.05). All the surveyed vegetarians struggled with vitamin B12 deficiency in their diet. In contrast, in the case of omnivores, almost half of those surveyed ate the recommended amounts according to the EFSA nutrition standard. This difference was statistically significant (*p* < 0.0.5).

**Table 8:** Comparison of providing the body with appropriate ingredients according to EFSA standards among vegetarians and omnivores.

Nutrients [PRI or AI], [*n* (%)]	Vegetarians (*n* = 22)		Omnivores (*n* = 22)		*p* Value
	Women (*n* = 18), Men (*n* = 4)		Women (*n* = 18), Men (*n* = 4)		
	AR	BR	AR	BR	
Fiber (g) [≥25 g]	19 (86.4)	3 (13.6)	12 (54.5)	10 (45.5)	0.0474 ^$^
Potassium (mg) [≥3500 mg]	10 (45.5)	12 (54.5)	11 (50)	11 (50)	0.7627
Calcium (mg) [18–24 years ≥ 1000 mg >25 years ≥ 950 mg]	7 (31.8)	15 (68.2)	8 (36.4)	14 (63.6)	0.7503
Phosphorus (mg) [≥550 mg]	21 (95.5)	1 (4.5)	22 (100)	0 (0)	1.0000
Magnesium (mg) [≥350 mg/≥300 mg]	17 (77.3)	5 (22.7)	16 (72.7)	6 (27.3)	0.7275
Iron (mg) [18–39 years ≥ 11 mg/≥16 mg ≥40 years ≥ 11 mg]	10 (45.5)	12 (54.5)	8 (36.4)	14 (63.6)	0.5393
Zinc (mg) [7.5–12.7 mg]	19 (86.4)	3 (13.6)	21 (95.5)	1 (4.5)	0.5597
Copper (mg) [≥6 mg/≥11.3 mg]	0 (0)	22 (100)	0 (0)	22 (100)	1.0000
Iodine (µg) [≥150 μg]	1 (4.5)	21 (95.5)	0 (0)	22 (100)	1.0000
Vitamin A (µg) [≥750 μg/≥650 μg]	21 (95.5)	1 (4.5)	21 (95.5)	1 (4.5)	0.4692
Vitamin D (µg) [≥15 μg]	0 (0)	22 (100)	1 (4.5)	21 (95.5)	1.0000
Vitamin E (mg) [≥13 mg/≥11 mg]	10 (45.5)	12 (54.5)	14 (63.6)	8 (36.4)	0.2245
Riboflavin (B_2_) (mg) [≥1.6 mg]	6 (27.3)	16 (72.7)	14 (63.6)	8 (36.4)	0.0142 ^$^
Vitamin B_6_ (mg) [≥1.7 mg/≥1.6 mg]	13 (59.1)	9 (40.9)	19 (86.4)	3 (13.6)	0.0905
Folic Acid (µg) [≥330 μg]	16 (72.7)	6 (27.3)	16 (72.7)	6 (27.3)	1.0000
Vitamin B_12_ (µg) [≥4 μg]	0 (0)	22 (100)	9 (40.9)	13 (59.1)	0.0027 ^$^
Vitamin C (mg) [≥110 mg/≥95 mg]	19 (86.4)	3 (13.6)	19 (86.4)	3 (13.6)	0.6604

Note: Data are presented as number (percentage) of participants, ^$^ *p*-value—statistically significant difference between vegetarians and omnivores, *p* < 0.05, Abbreviations: PRI—Population Reference Intake, AI—Adequate Intake, AR—According to References, BR—Below References.

The participants were also asked whether and what kind of supplementation they use (Table 9). Despite the lack of significance, more vegetarians used supplementation than omnivores (*p* > 0.05). The most commonly used supplements among vegetarians were vitamin D, vitamin B_12_, and magnesium. Significant differences were observed for vitamin B_12_ and iron (*p* < 0.05). In both cases, no omnivore supplemented these nutrients.

**Table 9:** Comparison of the supplements used by the surveyed vegetarians and omnivores.

Supplementation [*n*, (%)]	Vegetarians (*n* = 22)	Omnivores (*n* = 22)	*p* Value
	Women (*n* = 18), Men (*n* = 4)	Women (*n* = 18), Men (*n* = 4)	
Yes	17 (77.3)	14 (63.6)	0.2805
No	5 (22.7)	8 (36.4)	
Magnesium	7 (31.8)	2 (9.1)	0.0507
Calcium	3 (13.6)	2 (9.1)	1.0000
Zinc	0 (0)	1 (4.6)	1.0000
Iron	5 (22.7)	0 (0)	0.0574
Vitamin B_12_	16 (72.4)	0 (0)	0.0000 ^$^
Vitamin B_6_	1 (4.6)	0 (0)	1.0000
Vitamin D	13 (59.1)	9 (40.9)	0.2265
Biotin	1 (4.6)	0 (0)	1.0000
Omega-3 fatty acids	5 (22.7)	1 (4.6)	0.1875

Note: Data are presented as number (percentage) of participants, ^$^ *p*-value—statistically significant difference between vegetarians and omnivores, *p* < 0.05.

### 3.3. Blood Analysis Results

Both groups were sent for a blood biochemical analysis for selected parameters to assess the participants’ health more accurately. These results are presented in Table 10 and Table 11. Table 10 shows the mean values and standard deviations for blood chemistry parameters. The *p*-values for all comparisons were greater than 0.05, indicating no significant differences between the blood parameters among vegetarians and omnivores. Therefore, it was decided to compare the results of the biochemical analysis of the blood of the examined persons with the reference values. The results showed no statistically significant differences between the group of vegetarians and omnivores for any of the studied parameters (*p* > 0.05). Despite this, it is worth noting that the blood samples of vegetarians below the reference standard significantly more for the following parametres than omnivores: iron, ferritin, and vitamin B12. In the case of glucose, slightly more omnivores presented a level above the norm compared to vegetarians. In the case of homocysteine, more than half of vegetarians exceeded the reference value. The serum vitamin D level was similar for both groups; most subjects had low vitamin D concentrations in the blood serum.

### 3.4. Body Composition Analysis and Anthropometric Measurements

The body composition results of the bioelectrical impedance analysis and anthropometric measurements of vegetarians and omnivores are presented in Table 12. Only a statistically significant difference in basal metabolic rate (BMR) was observed between the two groups. Vegetarians, on average, presented a higher BMR compared to omnivores (*p* < 0.05). The other parameters had no statistically significant differences between vegetarians and omnivores (*p* > 0.05). Despite this, it is worth noting that vegetarians had lower body weight than omnivores, and a lower waist circumference, FM, FM%, and VAT. They had slightly less FFM than omnivores and lower total water content. The phase angle between the two groups is similar but somewhat lower for vegetarians.

Differences in BMI are insignificant; however, among the omnivores, more subjects struggled with excessive body weight, i.e., overweight or obesity, compared to the surveyed vegetarians. Detailed BMI results are presented in Table 13.

**Table 12:** Comparison of BIA body composition analysis results and anthropometric measurements of vegetarians and omnivores.

Parameter [$\overset{-}{x}$ ± SD]	Vegetarians (*n* = 22)	Omnivores (*n* = 22)	*p* Value
	Women (*n* = 18), Men (*n* = 4)	Women (*n* = 18), Men (*n* = 4)	
Body weight (kg)	67.29 ± 14.48	72.64 ± 16.56	0.2609
Growth (m)	1.68 ± 0.08	1.66 ± 0.08	0.3795
BMI (kg/m^2^)	24.02 ± 5.25	26.6 ± 6.1	0.1771
WC (cm)	79.73 ± 11.64	86 ± 26	0.7159
FM (kg)	20.23 ± 10.1	24.06 ± 12.53	0.4180
FM (%)	26.67 ± 9.44	31.9 ± 11.9	0.1697
FFM (kg)	47.08 ± 8.9	48.58 ± 11.01	0.8418
FMI (kg/m^2^)	7.33 ± 3.87	8.99 ± 4.91	0.4180
FFMI (kg/m^2^)	16.69 ± 2.3	17.61 ± 2.88	0.2505
Muscle mass (kg)	21.51 ± 5.12	22.66 ± 6.72	0.4180
Total body water content (L)	34.6 ± 6.56	35.57 ± 8.28	0.9719
Phase angle (percentile)	5.17 ± 0.58	5.37 ± 0.94	0.7335
VAT (L)	0.8 ± 0.7	1.04 ± 1.08	0.4113
PAL	1.58 ± 0.22	1.56 ± 0.19	0.8326
BMR (kcal/day)	1499.2 ± 242.9	1319.2 ± 321.4	0.0116 ^$^

Note: Each value is the mean ± standard deviation, ^$^ *p*-value—statistically significant difference between vegetarians and omnivores, *p* < 0.05, Abbreviations: BMI (body mass index), WC (waist circumference), FM (fat mass), FFM (fat-free mass), FMI (fat mass index), FFMI (fat-free mass index), VAT (visceral adipose tissue), PAL (physical activity level), BMR (basal metabolic rate).

## 4. Discussion

This study aimed to compare the health status of vegetarians to omnivores, assuming that people following a vegetarian diet for a long time will be characterized by better health. In recent years, there have been several studies comparing vegetarians and omnivores. However, none of these studies were as comprehensive as ours. Some studies focused exclusively on women [18], while others lacked anthropometric analysis [19,20,21,22]. Some studies included short-term vegetarians with limited blood analysis [23]. The study most similar to ours was conducted on a group of German vegetarians [24]. If there are studies concerning Polish vegetarians, they are also not as comprehensive and thorough [25,26,27]. For example, a recently published research on Polish vegetarians used questionnaires without body composition and blood biochemical analysis [26]. To obtain our results, we used a blood biochemical analysis, BIA body composition analysis, and an analysis of diet and eating habits. To our knowledge, there has yet to be a vegetarian study combining these health assessment methods.

The KomPAN-validated questionnaire for examining dietary views and patterns, developed by Polish scientists from the Polish Academy of Sciences [16], was used to learn about the nutritional habits of a given group and their opinions on nutrition. Thanks to it, we found out what eating habits and lifestyles characterize the surveyed vegetarians and omnivores. The eating habits we asked the respondents about concerned the number of meals consumed daily, the regularity of eating meals, and the frequency of snacking. Most vegetarians consumed four meals daily, and slightly fewer ate three. In the case of omnivores, a similar number of subjects ate four and three meals. A significant percentage of vegetarians ate meals regularly during the day. The Gacek study on 118 Polish vegetarians checked the number and regularity of meals eaten [25]. The results were consistent with our research. Most vegetarians ate three meals (55.8%), with slightly fewer (37.2%) consuming 4–5 meals.

The difference between vegetarians and omnivores regarding snacking between meals was significant. Vegetarians snacked less between meals than omnivores, which was a statistically significant difference. Similar results were presented by Kwiatkowska et al. [26]. According to their study conducted during the COVID-19 pandemic on Polish vegetarians, more omnivores (over 50%) snacked between meals than vegetarians. Also, in the study by Storz et al., more omnivores had ready access to and consumed snacks between meals than vegetarians [28]. It can be concluded that vegetarians present a healthier way of eating because, in most cases, they do not have the habit of snacking between meals, which does not lead to excessive overeating and the uncontrolled consumption of unplanned meals.

The present study shows that over 30% of vegetarians did not use any spreadable fat. Vegetarians who used spreadable fat most often chose margarine or butter. Vegetarians preferred vegetable oil, including olive oil, for frying. High consumption of fried foods may be associated with a higher risk of high blood pressure and being overweight. However, several factors impact the increasing risk of disease: the choice of fat for frying, the technique used, time and temperature. Other studies confirm that extra virgin olive oil reduces the risk of cardiovascular diseases and weight gain and recommends using olive oil for frying [29].

Despite using appropriate fat for frying, the surveyed vegetarians make dietary mistakes by sweetening hot drinks and adding salt to food. More than 50% of the surveyed vegetarians more often sweeten hot drinks with one or more teaspoons of sugar, while omnivores sweeten much less frequently. Similarly, in the case of salting, more than 50% of vegetarians confirm adding salt to food. Both relationships were statistically significant (*p* < 0.05). In the study by Marciniak et al., the vast majority—i.e., over 60% of the vegetarians questioned—did not add salt to ready meals. The situation was similar with the sweetening of hot drinks. A significant percentage (as high as 70%) of vegetarians declared they did not sweeten hot beverages [27]. The discrepancy in these results may be related to the fact that in our study, the group of vegetarians was smaller than the Marciniak study group (*n* = 390) [27].

Our study analyzed the frequency at which various food products were consumed, including grain products, vegetables, fruits, fast food, fried foods, water, assorted juices and sweetened drinks, energy drinks, and alcohol. Statistical differences between the frequency of consumption by vegetarians and omnivores were found in the case of legumes—much more often consumed by vegetarians, canned vegetables, and pickles, which were also much more often consumed by vegetarians, and in drinking vegetables or vegetables and fruit juices as well as alcohol. Consumption of this drink type was statistically higher among vegetarians than omnivores. Despite the higher consumption of alcohol by vegetarians, it can be concluded that their diet complied with the principles of healthy eating. They consumed whole grain products, fruits, and vegetables sufficiently often, and much less fast food, energy drinks, sweetened carbonated and non-carbonated drinks, and sweets. Such conclusions have also been drawn by other researchers [27,30,31].

To take a holistic approach to assessing vegetarians’ health status and lifestyle, the subjects were asked about smoking, hours of sleep on weekdays and weekends, the amount of time spent in front of the TV and computer monitor, and physical activity on weekdays and weekends. The vast majority of both vegetarians and omnivores were non-smokers. In addition, both groups presented similar hours of sleep during the week and at weekends and equal amounts of time spent in front of the television and computer monitors. Despite similar PAL rates in vegetarians and omnivores, there was a clear difference in leisure time: vegetarians were more likely to spend this time in physical activity than omnivores. This could mean that the vegetarians surveyed were more aware of the healthy lifestyle principles. Physical activity is important for maintaining good health [32]. The similarity of the PAL rate in both groups is related to the fact that it referred to the average total physical activity performed during the day, including working time, where the physical activity performed was not a choice, but a necessity related to the nature of the job.

The results of the subject’s subjective diet assessments suggest that people on a vegetarian diet tend to assess their health better than their peers who eat meat. Also, they believed that they consumed appropriate and healthy levels of nutrition. Researchers from the University of Economics in Wrocław, Poland, came to similar conclusions. As in our study, vegetarians and omnivores participated. Vegetarians (*n* = 190) constituted the majority of subjects who were satisfied with their diet. Over 66% rated their diet as “very good” and 25% as “good” [33].

Body composition analyses using the BIA method were performed to assess the health of vegetarians accurately. Vegetarians had lower BMI, body weight, and waist circumference than omnivores. The differences were not statistically significant, confirming the results of Saintila et al. [34]. In their study, vegetarians also demonstrated better results in these anthropometric parameters. Teixeira et al. [35] compared the nutritional status of vegetarians and omnivores aged 35 to 65 living in Brazil. The results indicated a higher risk of being overweight among omnivores. Similar conclusions were drawn in a study based on an anthropometric analysis of men following a vegetarian and non-vegetarian diet. The authors of this study also confirmed a lower risk of being overweight among vegetarians [36]. Despite the lack of statistical significance, we would like to highlight one detail in our study. Over 30% of the vegetarians we surveyed struggled with being overweight, and almost 10% with obesity. There are studies stating that despite the general principles of a vegetarian diet, related to giving up products of animal origin, including more vegetables and fruits in the daily menu, many vegetarians lose themselves in processed foods and snacks, causing uncontrolled weight gain, leading to overweight and obesity [37]. It is worth looking at this problem and researching in this direction on a larger population.

As in the case of the above parameters, the content of adipose tissue and visceral adipose tissue was lower among vegetarians than omnivores. Statistically significant differences in the adipose tissue content of both groups were observed in a study by Gan et al. [38]. Vegetarians had significantly less body fat than omnivores. Surprisingly, vegetarians also had less lean tissue, muscle mass, and total body water. We also decided to measure the phase angle of the test subjects. The biological significance of the phase angle has yet to be fully discovered, but it is considered in assessing the health of body cells [39]. A higher phase angle value indicates that the cells are in a better condition. Existing studies suggest that the range of population norms for healthy subjects’ phase angle is 5–7°, and below 5° indicates malnutrition [40,41]. The phase angle was satisfactory among vegetarians and omnivores, proving the appropriate condition of the cells of the examined subjects.

Vegetarians have a significantly higher basal metabolic rate. This also translated into the seven-day average intake of energy. Vegetarians consumed more energy than omnivores, although the difference was not statistically significant. Unfortunately, we have not found studies confirming these results and the thesis that vegetarians have a greater demand for resting energy than omnivores. Therefore, it is worth considering this issue and researching this area.

Moreover, most existing studies of a similar nature indicate an inverse correlation. In one, the differences in the consumption of selected nutrients and energy supply with food among vegetarians and omnivores were examined. Compared to omnivores, the energy value of dishes eaten by vegetarians was significantly lower [42]. In turn, in the study by Gan et al., the difference between the energy supplied by food among vegetarians (2070 ± 570 kcal) and omnivores (2120 ± 585 kcal) was not statistically significant [38].

Mean carbohydrate and protein intakes did not differ significantly between vegetarians and omnivores. Omnivores consumed slightly more protein than vegetarians, while vegetarians consumed more carbohydrates. The same results were obtained in the study by Gan et al. [38]. This was undoubtedly related to vegetarians’ high fiber intake, which was statistically significantly different from the amount of fiber consumed by omnivores. The larger dietary fibre intake is due to the high consumption of wholegrain cereals, legumes, and vegetables, which was also indicated in the answers to the KOMPAN questionnaire. Consuming adequate dietary fiber is critical in preventing many diet-related diseases, including cardiovascular diseases, cancer, type 2 diabetes, and obesity [43,44].

Compared to omnivores, vegetarians consumed statistically significantly less phosphorus, iodine, and riboflavin in their food. Many factors can affect phosphorus concentration in the human blood, including parathyroid hormone, fibroblast growth factor 23, vitamin D, and diet [45]. Therefore, it is unsurprising that omnivores presented a much higher level of phosphorus intake than vegetarians because phosphorus is mainly found in food of animal origin: milk, red meat, and poultry. Phosphorus deficiency is extremely rare in a healthy population [46]. It is also believed that excessive phosphorus consumption may adversely affect human health—e.g., through calcification of vessels and, consequently, the development of atherosclerosis [47]. Iodine is responsible for the synthesis of thyroid hormone. Before introducing iodine fortification programs, the incidence of iodine deficiency was widespread worldwide [48]. Iodine deficiency can be associated with fetal miscarriage during pregnancy, developmental defects, endemic cretinism, impaired mental function, delayed physical development, and an increased risk of hyperthyroidism [49]. Iodine deficiencies have been reported among Norwegian vegetarians and vegans. As many as half of the examined subjects did not consume the recommended amount of iodine according to the EAR (Estimated Average Requirement) standard [50]. In our study, only one person was iodine deficient. Others had adequate amounts of this ingredient in their diet. Riboflavin deficiency, found in almost 30% of the vegetarians we studied, may be associated with increased homocysteine levels by reducing the metabolism of other B vitamins [51]. A similar percentage of vegetarians was deficient in riboflavin, according to a study by Majchrzak et al. and was more significant compared to omnivores, of whom 10% were iodine deficient [51]. Folic acid is a vitamin found mainly in yeast, sprouts, legumes, green vegetables, and the liver [52]. Among vegetarians, over 70% of the subjects were deficient in folic acid.

Interestingly, the same number of omnivores did not have enough folic acid in their diet. Different results were obtained by Schüpbach et al. [53]. According to their study, vegetarians presented the highest intake of folic acid, followed by vegans, while omnivores recorded the lowest intake. High levels of folic acid have also been demonstrated in vegetarians from Spain [54]. Due to the discrepancies between our results and the available literature, it is also worth making a more extensive analysis of the status of folic acid in the diet of Polish vegetarians.

The use of supplements is becoming increasingly popular among various population groups. A vegetarian diet rises to be deficient, and supplementation may be particularly justified [55]. Based on the results of our study, we conclude that vegetarians benefit from supplementation (70% of the surveyed group). Similar results were obtained by Grzelak et al. [56]. In their case, over 70% of the surveyed vegetarians used supplementation. Most often, they took preparations with vitamin D and B12, which was also reflected in the results of our study. In addition, we noted that vegetarians were more likely to take magnesium supplements.

When designing this study, we were convinced that the blood analysis results of the examined subjects would help us assess their health condition and show statistically significant differences for specific parameters. One method of assessing cardiovascular disease risk is blood analysis for Total-cholesterol, HDL, TG, and LDL. One of the main causative and modifiable risk factors for atherosclerotic cardiovascular disease is lipoproteins, among which low-density lipoproteins (LDL) are the most numerous [57]. High levels of triglycerides are also associated with cardiovascular disease, metabolic syndrome, and pancreatitis. Elevated levels of LDL cholesterol and TG may be affected by high SFA intake [58]. In our study, SFA intake among vegetarians was slightly lower than among omnivores. In turn, low levels of HDL cholesterol may also contribute to the occurrence of cardiovascular diseases [59]. In our study, we analyzed the lipid profile of vegetarians and compared them to a control group of omnivores. In the case of HDL, more than 90% of vegetarians and omnivores exceeded the minimum reference values, while these differences were not statistically significant.

Past studies on vegetarians and omnivores evaluating the concentration of this cholesterol fraction also showed the same results [34,60]. De Biase’s et al. study on vegetarians and omnivores was designed to compare the TG, HDL, LDL, and total cholesterol values in both groups. There was a significant difference between the groups for total cholesterol, LDL, and TG, with these values being higher in omnivores compared to vegetarians and lower the more restrictive the vegetarian diet was [61]. In our study, in the case of LDL and TG among vegetarians and omnivores, most subjects had concentrations of both parameters in line with the reference values. Different results were presented by Saintila et al. [34]. The vegetarians who participated in their study had higher blood LDL levels than omnivores. Researchers explained this by the possibility that they consumed more processed carbohydrates compared to omnivores.

High glucose levels may contribute to hyperglycemia and, consequently, to insulin resistance and diabetes [62]. Blood analysis showed that most vegetarians did not have high blood glucose levels. Compared to the results of omnivores, this was not statistically significant. Still, it may be confirmed that a vegetarian diet could be a preventive measure in the context of diabetes [63]. Moreover, another study demonstrates that a properly balanced plant-based diet, particularly a vegan one, improves diabetes management by reducing blood glucose levels and leading to clinical improvement in people with type 2 diabetes [64]. Lee et al. published the results of a study that checked whether vegetarian and vegan diets were beneficial in terms of glycemic control in patients with type 2 diabetes. They found that both diets—a less restrictive vegetarian and a more restrictive vegan diet led to a reduction in HbA1c (glycated hemoglobin). However, glycemic control was better with a vegan diet [65]. In our study, we did not analyze glycated hemoglobin, which is why it is a good starting point for further research on Polish vegetarians in this area.

Albumin is the most abundant protein in human serum. It is used to indicate malnutrition [66]. Therefore, we decided to test its concentration in the blood of the surveyed vegetarians and compare it to omnivores. The results suggest that the nutritional status of vegetarians and omnivores was at the right level. The same goes for total protein. A few vegetarians presented a lower concentration of this parameter than the reference range. Sylvie et al. obtained similar results [67]. By analyzing blood samples for albumin concentrations in 101 vegetarians, they observed that none had below-normal albumin levels.

The blood analysis conducted in our study was intended to indicate deficiencies of minerals and vitamins associated with a vegetarian diet. Iron is one such mineral. Plant-based diets that exclude meat and animal products may have lower iron levels because they only provide non-heme iron, which has low bioavailability [68]. In addition, vegetarian diets are rich in iron absorption inhibitors, including phytates and polyphenols [69]. Iron deficiency can lead to anemia, activate bone resorption, and affect the immune system [69,70]. In our study, we analyzed the concentration of iron and ferritin in the blood. In the case of ferritin, most vegetarians had an appropriate level. Iron was within the norm in more than 70% of vegetarians, with almost 20% having concentrations exceeding the reference range. Ferritin and iron concentrations were normal among almost all omnivores. Similar results were obtained by Schüpbach et al. while researching Swiss vegetarians [53]. At the same time, it is worth noting that more than half of the surveyed vegetarians consumed adequate amounts of iron. Similar results were obtained by Slywitch et al. [71]. In a study of over 1300 people, there was no difference in iron deficiency incidence in vegetarians and omnivores [71]. In our study, more than 20% of the surveyed vegetarians consciously supplement this mineral, which may also translate into better serum iron levels in these tested subjects.

Vitamin B12 is essential for cell division and is involved in blood formation and the proper functioning of the nervous system [72]. Vitamin B12 deficiency can cause abnormal neurological symptoms and mood and concentration disorders, leading to macrocytic anemia [72,73]. Because they avoid meat products, people on a vegetarian diet are particularly at risk of deficiency of this vitamin because vitamin B12 is mainly found in products of animal origin [72]. This was also the case with the vegetarians we studied. The vast majority presented a concentration of vitamin B12 in the blood that fell below reference values.

Moreover, an insufficient supply of vitamin B12 in food was also noted. None of the tested vegetarians consumed adequate amounts of vitamin B12 in their food. Despite deficiencies, most vegetarians knew the risk of vitamin B12 deficiency, as over 70% of respondents supplemented it. Existing studies also confirm the notorious problem of vitamin B12 deficiency among subjects following a plant-based diet [74]. In addition, vitamin B12 deficiency can cause an increase in homocysteine levels in the blood. In our study, more than half of the vegetarians had elevated serum homocysteine levels. Almost without exception, studies comparing blood homocysteine concentrations of vegetarians and omnivores indicate higher total homocysteine concentrations among vegetarians and the highest concentrations among vegans compared to omnivores [75]. Despite scientific evidence demonstrating the preventive effect of plant-based diets on cardiovascular disease [4,6,76], homocysteine accumulation is independently associated with the risk of cardiovascular disease or endothelial dysfunction [77]. According to a systematic review and meta-analysis from 2008, for each 5 μmol/L increase in homocysteine, there was a 20% increase in the risk of ischemic heart disease [78].

Also, the vitamin D concentration in the blood serum was lower among vegetarians than omnivores. The same applies to the intake of vitamin D with food. Such results have already been confirmed in previous studies [79,80]. Long-term vitamin D deficiency may be associated with osteomalacia, osteoporosis, and rickets. In addition, it can cause neurological diseases, ischemic heart disease, type 2 diabetes, and autoimmune diseases [81]. Therefore, if it is impossible to consume vitamin D in the diet, the body should be exposed to sunlight on sunny days, thanks to which vitamin D will be formed in the skin, and vitamin D supplementation should also be considered [82]. Over half of the vegetarians, we surveyed were systematically supplemented with vitamin D.

## 5. Conclusions

To obtain comprehensive results on vegetarians’ health, eating habits, and dietary style, we utilized various tools that allowed us to conduct a thorough analysis, despite the relatively small number of respondents. Polish vegetarians exhibit similar health statuses and tend towards better health than omnivores. They demonstrate improved body composition indices as determined by the BIA method. Biochemical blood analysis revealed no significant differences in blood parameters between vegetarians and omnivores. However, vegetarians were found to have specific deficiencies, such as vitamin B_12_, vitamin D, and elevated homocysteine levels, which may be attributed to a lack of vitamin B_12_ intake.

Moreover, most surveyed vegetarians reported taking supplements of minerals and vitamins that are difficult to obtain solely from a plant-based diet, indicating their heightened awareness of the risk of nutritional deficiencies associated with such dietary choices. Vegetarians also exhibit a better nutritional status than omnivores and adhere to accepted dietary principles, consuming higher quantities of vegetables, fruits, and cereals while consuming fewer sugary drinks, energy drinks, and fast-food meals. Additionally, the lifestyle of vegetarians can be characterized as healthy due to their greater willingness to engage in physical activity compared to omnivores and adequate sleep. Although our study was limited by the small sample size, conducting a more extensive analysis on a larger population of Polish vegetarians would be valuable.

## Figures and Tables

**Table 1:** Comparison of eating habits of vegetarians and omnivores.

Eating Habits [*n* (%)]	Vegetarians (*n* = 22)	Omnivores (*n* = 22)	*p* Value
	Women (*n* = 18), Men (*n* = 4)	Women (*n* = 18), Men (*n* = 4)	
***Number of meals eaten***			
Five meals or more	4 (18.2)	4 (18.2)	0.644
Four meals	10 (45.5)	8 (36.4)	
Three meals	8 (36.4)	9 (40.9)	
Two meals	0 (0)	1 (4.6)	
***Eating meals at regular times of the day***			
Yes, all	5 (22.7)	2 (9.1)	0.361
Yes, but only some	11 (50)	15 (68.2)	
No	6 (27.3)	5 (22.7)	
***Eating between meals***			
Several times a day	5 (22.7)	3 (13.6)	0.026 ^$^
Once a day	4 (18.2)	5 (22.7)	
A few times a week	5 (22.7)	11 (50)	
Once a week	5 (22.7)	1 (4.6)	
1–3 times a month	0 (0)	2 (9.1)	
Never	3 (13.6)	0 (0)	
***Type of spreadable fat used***			
Mayonnaise	0 (0)	1 (4.6)	0.119
Margarine	6 (27.3)	5 (22.7)	
Butter	5 (22.7)	5 (22.7)	
Mix butter with margarine	0 (0)	3 (13.6)	
Hummus	0 (0)	1 (4.6)	
Vegan “butter”	2 (9.1)	0 (0)	
I use different fats	2 (9.1)	0 (0)	
I don’t	7 (31.8)	7 (31.8)	
***The kind of frying fat used***			
Vegetable oil (including olive oil)	17 (77.3)	14 (63.6)	0.119
Butter	4 (18.2)	3 (13.6)	
Coconut oil	1 (4.6)	0 (0)	
Lard	0 (0)	1 (4.6)	
I use different fats	0 (0)	3 (13.6)	
I don’t use any fat for frying	0 (0)	1 (4.6)	
***The sweetening of hot drinks***			
Yes, I sweeten it with two or more teaspoons of sugar (or honey)	5 (22.7)	6 (27.2)	0.023 ^$^
Yes, I sweeten it with one teaspoon of sugar (or honey)	7 (31.8)	1 (4.6)	
Yes, I use sweeteners (low-energy sweeteners)	0 (0)	3 (13.6)	
No	10 (45.5)	12 (54.6)	
***Adding salt to meals***			
Yes, I add salt to most dishes	5 (22.7)	0 (0)	0.018 ^$^
Yes, but only sometimes	8 (36.4)	8 (36.4)	
No	9 (40.9)	14 (63.6)	

Note: Data are presented as number (percentage) of participants, ^$^ *p*-value—statistically significant difference between vegetarians and omnivores, *p* < 0.05.

**Table 10:** Biochemical parameters [$\overset{-}{x}$ ± SD] among vegetarians and omnivores.

Parameter	Vegetarians (*n* = 22)	Omnivores (*n* = 22)	*p* Value
	Women (*n* = 18), Men (*n* = 4)	Women (*n* = 18), Men (*n* = 4)	
Glucose (mg/dL)	92.67 ± 6.95	94.14 ± 7.53	0.7692
Calcium (mg/dL)	9.35 ± 0.29	9.32 ± 0.23	0.7016
Total cholesterol (mg/dL)	178.81 ± 31.83	185.45 ± 26.37	0.4550
HDL (mg/dL)	65 ± 16.18	60.82 ± 13.26	0.3535
LDL (mg/dL)	95.23 ± 32.26	105.18 ± 27.13	0.1733
TG (mg/dL)	91.7 ± 44.84	96.85 ± 45.35	0.6220
Total protein (g/dL)	6.9 ± 0.5	7.03 ± 0.4	0.3538
Albumin (g/dL)	45.55 ± 2.01	46.33 ± 2.33	0.2418
Iron (µg/dL)	104.21 ± 45.92	90.4 ± 26.3	0.4596
Ferritin (ng/mL)	39.17 ± 26.53	76.55 ± 68.45	0.0500
Vitamin B_12_ (pg/mL)	446.74 ± 208.31	450.16 ± 187.85	0.9547
Vitamin D_3_ (ng/mL)	26.29 ± 11.62	28.59 ± 13.03	0.6055
Homocysteine (µmol/L)	13.11 ± 3.41	11.68 ± 3.18	0.1572
Leukocytes (×10^3^/µL)	5.88 ± 1.55	5.74 ± 1.52	0.7731
Erythrocytes (×10^6^/µL)	4.63 ± 0.3	4.73 ± 0.57	0.4458
Hemoglobin (g/dL)	13.8 ± 1.04	13.92 ± 1.48	0.7485
Platelets (×10^3^/µL)	251.52 ± 55.02	239.91 ± 52.89	0.9158

Note: Each value is the mean ± standard deviation. Abbreviations: HDL (high-density lipoprotein), LDL (low-density lipoprotein), TG (triglycerides).

**Table 11:** Comparison of the results of biochemical parameters with reference values of study groups.

Parameter	Reference Range		Vegetarians (*n* = 22)			Omnivores (*n* = 22)			*p* Value
	Men	Women	Women (*n* = 18), Men (*n* = 4) [*n* (%)]			Women (*n* = 18), Men (*n* = 4) [*n* (%)]			
			BR	AR	ER	BR	AR	ER	
Glucose (mg/dL)	70–99		0 (0)	19 (86)	3 (14)	0 (0)	17 (77)	5 (23)	0.6958
Calcium (mg/dL)	8.6–10.0		0 (0)	21 (95)	1 (5)	0 (0)	22 (100)	0 (0)	1.0000
Total-chol (mg/dL)	115–190		0 (0)	14 (63)	8 (36)	0 (0)	14 (64)	8 (36)	1.0000
HDL (mg/dL)	>40	>45	0 (0)	1 (5)	21 (95)	0 (0)	2 (9)	20 (91)	1.0000
LDL (mg/dL)	<115		0 (0)	15 (68)	7 (32)	0 (0)	15 (68)	7 (32)	1.0000
TG (mg/dL)	35–150		0 (0)	19 (86)	3 (14)	0 (0)	19 (86)	3 (14)	0.6604
Total protein (g/dL)	6.40–8.30		3 (14)	19 (86)	0 (0)	0 (0)	22 (100)	0 (0)	0.2316
Albumin (g/dL)	35–52		0 (0)	22 (100)	0 (0)	0 (0)	22 (100)	0 (0)	1.0000
Iron (µg/dL)	59–158	37–145	2 (9)	16 (73)	4 (18)	0 (0)	21 (95)	1 (5)	0.0679
Ferritin (ng/mL)	30–400	13–150	2 (9)	20 (91)	0 (0)	1 (5)	19 (86)	2 (9)	0.4218
Vitamin B_12_ (pg/mL)	160–800		2 (9)	18 (82)	2 (9)	1 (5)	19 (86)	2 (9)	0.8324
Vitamin D_3_ (ng/mL)	30–50		15 (68)	6 (27)	1 (5)	13 (59)	7 (32)	2 (9)	0.9040
Homocysteine (µmol/L)	<12		0 (0)	9 (41)	13 (59)	0 (0)	14 (64)	8 (36)	0.1295
Leukocytes (×10^3^/µL)	4–10		1 (5)	21 (95)	0 (0)	3 (14)	19 (86)	0 (0)	0.6000
Erythrocytes (×10^6^/µL)	4.5–6.0	3.8–5.4	0 (0)	22 (100)	0 (0)	1 (5)	19 (86)	2 (9)	0.1120
Hemoglobin (g/dL)	14–18	12–16	2 (9)	20 (91)	0 (0)	3 (14)	17 (77)	2 (9)	0.4282
Platelets (×10^3^/µL)	130–400		0 (0)	21 (95)	1 (5)	0 (0)	22 (100)	0 (0)	1.0000

Note: Data are presented as number (percentage) of participants, Abbreviations: BR—below references, AR—according to references, ER—exceeded references. The reference ranges come from the external laboratory where the blood analysis was performed.

**Table 13:** Comparing the BMI of vegetarians with omnivores.

BMI	Vegetarians (*n* = 22)	Omnivores (*n* = 22)	*p* Value
	Women (*n* = 18), Men (*n* = 4)	Women (*n* = 18), Men (*n* = 4)	
Obesity	2 (9.1)	8 (36.4)	0.16724
Overweight	6 (27.3)	4 (18.2)	
Correct Body Weight	12 (54.6)	8 (36.4)	
Underweight	2 (9.1)	2 (9.1)	

Note: Data are presented as number (percentage) of participants, Abbreviations: BMI (Body Mass Index).

## Data Availability

The data presented in this study are available on request from the corresponding author. The data are not publicly available due to restrictions concerning privacy.
